# Prevalence of type 2 diabetes mellitus and its predictive factors in Italy: a comparison between HIV-infected and uninfected subjects

**DOI:** 10.1186/1758-2652-13-S4-P230

**Published:** 2010-11-08

**Authors:** L Galli, S Salpietro, G Pellicciotta, A Galliani, PM Piatti, H Hasson, M Guffanti, N Gianotti, A Bigoloni, A Lazzarin, A Castagna

**Affiliations:** 1 San Raffaele Scientific Institute, Infectious Diseases, Milan, Italy; 2 San Raffaele Scientific Institute, Occupational Health, Milan, Italy; 3 H San Raffaele Resnati, Milan, Italy; 4 San Raffaele Scientific Institute, Internal Medicine Department, Milan, Italy

## Purpose of the study

We determined the prevalence of type-2 diabetes mellitus (DM) in HIV infected (HIV+) and uninfected (HIV-) subjects.

## Methods

Cross-sectional analysis on HIV+ patients (pts), aged >18 years [median(IQR): 46(41-51)], who attended the Infectious Diseases Department of the San Raffaele Scientific Institute, alive or lost or dead after 2007 and HIV- subjects, healthy workers, aged >18 years [median(IQR): 47(40-53)], evaluated between 2007-2008, all over Italy (15 Italian regions), in a campaign for the assessment of cardiovascular risk factors, promoted by the Occupational Medicine of the H San Raffaele Resnati. Logistic regression used to determine the risk of DM; odds ratios(OR) and its 95% confidence intervals reported.

## Results

4249 HIV+ (3248 males) and 9148 HIV- (7052 males) individuals. HIV+ pts had a higher prevalence of DM than HIV- [N=172 (4.1%) vs N=225 (2.5%), p<0.0001; OR=1.68 (1.37-2.05)]. Prevalence of DM was still higher among HIV+ than HIV- after controlling for body mass index (BMI) [<25: 3.2% vs 1.1%; 25-29.9: 2.9% vs 3.1%; >=30: 12.7% vs 7.8%; OR=1.79(1.29-2.50)], age [<=50 years old (yrs): 1.7% vs 1.2%; >50yrs: 10.8% vs 4.9%; OR=2.02(1.65-2.49)] or gender [Females: 2.7% vs 1.1%; Males: 4.5% vs 2.9%; OR=1.69(1.38-2.06)] or both factors [Females<=50yrs: 0.9% vs 0.8%; Females >50yrs: 11.1% vs 1.8%; Males<=50yrs: 2.0% vs 1.3%; Males>50yrs: 10.8% vs 5.6%; OR=2.02(1.64-2.48)].

Among subjects with DM, HIV+ pts were significantly different compared to HIV- as follows: were older(p<0.0001), had a lower BMI(p<0.0001), lower cholesterol(p<0.0001), lower HDL-cholesterol(p<0.0001), lower fasting glucose(p<0.0001) and higher triglycerides (p=0.019). HIV+ and HIV- pts with DM were similar with respect to LDL-cholesterol, systolic and diastolic pressure and smoking status. After adjustment for age (<=50yrs, >50yrs), gender, BMI (<25, 25-29.9, >=30), cholesterol, HDL- and LDL-cholesterol, triglycerides and hypertension (yes vs no), HIV+ pts had a higher risk of diabetes (OR=1.71(1.02-2.86), p=0.043). Increasing age [>50yrs vs <=50yrs: OR=4.10(3.01-5.59), p<0.0001] or BMI [25-29.9 vs <25: OR=1.87(1.28-2.74); >=30 vs <25: OR=4.67(3.08-7.10); overall effect: p<0.0001] were also predictive factors of a greater risk of DM.

## Conclusions

Our findings suggest an increased prevalence of type-2 diabetes in HIV+ than HIV- subjects which was almost doubled in HIV+ than HIV- and up to 4-fold higher among obese subjects or those aged>50 years.

